# Identification of a Highly Conserved Hypothetical Protein TON_0340 as a Probable Manganese-Dependent Phosphatase

**DOI:** 10.1371/journal.pone.0167549

**Published:** 2016-12-01

**Authors:** Young-Sik Sohn, Seong-Gyu Lee, Kwang-Hoon Lee, Bonsu Ku, Ho-Chul Shin, Sun-Shin Cha, Yeon-Gil Kim, Hyun Sook Lee, Sung-Gyun Kang, Byung-Ha Oh

**Affiliations:** 1 Department of Biological Sciences, KAIST Institute for the Biocentury, Korea Advanced Institute of Science and Technology, Daejeon, Korea; 2 Disease Target Structure Research Center, Korea Research Institute of Bioscience and Biotechnology, Daejeon, Korea; 3 Department of Chemistry and Nano Science, Ewha Womans University, Seoul, Korea; 4 Pohang Accelerator Laboratory, Pohang University of Science and Technology, Pohang, Kyungbuk, Korea; 5 Marine Biotechnology Research Center, Korea Institute of Ocean Science & Technology, Ansan, Korea; Russian Academy of Medical Sciences, RUSSIAN FEDERATION

## Abstract

A hypothetical protein TON_0340 of a *Thermococcus* species is a protein conserved in a variety of organisms including human. Herein, we present four different crystal structures of TON_0340, leading to the identification of an active-site cavity harboring a metal-binding site composed of six invariant aspartate and glutamate residues that coordinate one to three metal ions. Biochemical and mutational analyses involving many phosphorous compounds show that TON_0340 is a Mn^2+^-dependent phosphatase. Mg^2+^ binds to TON_0340 less tightly and activates the phosphatase activity less efficiently than Mn^2+^. Whereas Ca^2+^ and Zn^2+^ are able to bind to the protein, they are unable to activate its enzymatic activity. Since the active-site cavity is small and largely composed of nearly invariant stretches of 11 or 13 amino acids, the physiological substrates of TON_0340 and its homologues are likely to be a small and the same molecule. The Mn^2+^-bound TON_0340 structure provides a canonical model for the ubiquitously present TON_0340 homologues and lays a strong foundation for the elucidation of their substrate and biological function.

## Introduction

*Thermococcus onnurineus* NA1 is a hyperthermophilic archaeon isolated from a deep sea hydrothermal vent area [1]. Although this organism possesses metabolic pathways for utilizing common organic compounds, its genome also encodes proteins that are involved in the oxidative utilization of CO as an energy and carbon source [2]. The organism was shown to grow on formate as a sole energy and carbon source by oxidizing formate into CO_2_ and transferring the formate-derived electrons to protons, thus generating H_2_ [3, 4]. The protein TON_0340 (268 amino acids) was identified in a transcriptome study aimed at identifying genes whose expression is enhanced when this organism was grown with CO as the sole carbon and energy source. The biological function of TON_0340 is unknown, and a *BLAST* database search shows that TON_0340 belongs to the DUF4392 superfamily (after domain of unknown function), the members of which are found in all three domains of life. The sequence similarity between the family members is remarkably high, as illustrated by a human protein C14orf159 (chromosome14 open reading frame 159; 621 amino acids), whose gene might be regulated by estrogen receptor α [5]. This protein contains a C-terminal domain that shares 36% sequence identity with TON_0340. While the ubiquitous presence and the high sequence homology suggest an important physiological role of the TON_0340 homologues, their biochemical or biological functions have not been characterized at all. In addition, these proteins are not homologous to any functionally annotated proteins.

To gain insights into the biochemical function of TON_0340 and its homologues, we determined the crystal structures of TON_0340 in four different forms; the apo form without a bound metal ion and the Mn^2+^-, Mg^2+^- or Ca^2+^-bound form, revealing that the protein has a highly conserved bi-metal binding pocket. TON_0340 exhibits low, but detectable phosphatase activity towards many different phosphate-containing compounds. We also show that the phosphatase activity depends on Mn^2+^. While Mg^2+^ could activate this enzyme activity, it is less efficient than Mn^2+^. Thus, this work identifies a family of evolutionary conserved Mn^2+^-dependent phosphatases.

## Materials and Methods

### Gene cloning, protein expression and purification

A DNA fragment encoding the full-length TON_0340 protein amplified from cells of *T*. *onnurineus* NA1 using the oligodeoxyribonucleotide primers GCGA**CATATG**CCGGAGATTCCGAAGGACTTCTTC (with *Nde*i site) and GCGA**GTCGAC**GAGGCCAGCGAGGTACTCCATAAGG (with *Hin*dIII site). The amplicon was cloned into pET22b-CPD 10H, a modified form of the pET22b plasmid (Novagen) to express a protein fused to (His)_10_-tagged CPD (cysteine protease domain) at the C-terminus [6]. The fusion protein was expressed in the *E*. *coli* strain BL21(DE3) RIPL (Novagen) at 310 K. Bacterial lysates were prepared by sonication in a solution composed of 20 mM Tris-HCl pH 7.5, 0.1 M NaCl and 5 mM β-mercaptoethanol (Buffer A). Cleared lysates were loaded on to a column packed with HisPur Cobalt Resin (Thermo) and washed with Buffer A containing additional 10 mM imidazole. On-gel auto-cleavage of (His)_10_-tagged CPD was performed by incubating the resin with Buffer A containing 100 μM phytate for 2 h at room temperature, which activates the protease activity of CPD. TON_0340 was eluted with Buffer A from the column as the unbound fraction and loaded onto HitrapQ HP column (GE Healthcare). TON_0340 was eluted with a 0.0–0.5 M linear gradient of NaCl in twenty column volumes, and the fractions containing TON_0340 were pooled and applied onto HiLoad 26/60 Superdex75 prep-grade column (GE Healthcare) equilibrated with a buffer solution composed of 20 mM Tris-HCl pH 7.5, 0.1 M NaCl and 1 mM dithiothreitol. The purified TON_0340 protein was concentrated to 9 mg ml^-1^ using an Amicon Ultra-10 (Millipore).

### Crystallization and data collection

A number of initial crystals were obtained by screening 480 different commercially available precipitant solutions at 295K by using a Mosquito liquid handling system (TTP Lab Tech). Optimized crystallization conditions were searched in the format of the hanging-drop vapor diffusion method. Crystals of the apo form of TON_0340 grew in a precipitant solution containing 0.1 M sodium cacodylate pH 6.5 and 1.0 M ammonium phosphate monobasic. In order to obtain crystals of TON_0340 bound to a specific metal ion, the protein was first dialyzed against Buffer A containing additional 20 mM ethylenediaminetetraacetic acid (EDTA) to remove any bound metal ions. Crystals of Mn^2+^-, Mg^2+^- or Ca^2+^-bound TON_0340 grew in a precipitant solution commonly containing 0.1 M sodium acetate pH 5.5, 16% 2-methyl-2,4-pentanediol and additional 130 mM MnCl_2_, 27 mM Mg(CH_3_COO^-^)_2_ or 140 mM CaCl_2_, respectively. A native data set for the apo form of TON_0340 was collected on a Rigaku R-AXIS IV^++^ area detector with monochromated Cu*K*α X-rays generated by a RU-200 rotating anode generator (Rigaku/MSC) operated at 90 mA and 50 kV, and data sets for Mn^2+^- or Ca^2+^-bound TON_0340 were collected using synchrotron X-ray radiation. All diffraction data were integrated and scaled with *HKL2000* [7].

### Structure determination

Using the structure of Zn^2+^-bound TON_0340 (PDB ID: 4FC5) [8] as the search model, the structures of the apo form and the Mn^2+^-, Mg^2+^- or Ca^2+^-bound form of TON_0340 were determined by the molecular replacement method using MolRep [9]. Model building and crystallographic refinement were performed using COOT [10] and CNS [11], and final structures were evaluated using PROCHECK [12]. The space group and the crystal packing interactions of the Mn^2+^-, Mg^2+^- or Ca^2+^-bound crystal forms were the same as those of the Zn^2+^-bound crystal form. Data collection and refinement statistics for the four crystals are summarized in Table 1. The atomic coordinates of the four TON_0340 structures together with the structure-factor files have been deposited in the Protein Data Bank under accession codes 5GKX (the apo form), 5GL4 (the Mn^2+^-bound form), 5GL3 (the Mg^2+^-bound form) and 5GL2 (the Ca^2+^-bound form). All structure figures were prepared with *PyMOL* (http://www.pymol.org).

**Table 1:** Data collection and structure refinement statistics.

	Apo	Mn^2+^-bound	Mg^2+^-bound	Ca^2+^-bound
X-ray source^a^	Cu anode	NW12A, PF	5C, PAL	5C, PAL
(wavelength, Å)	(1.5418)	(1.0000)	(1.0000)	(1.0000)
Space group	P2_1_2_1_2_1_	P4_3_2_1_2	P4_3_2_1_2	P4_3_2_1_2
Unit cell dimension				
a, b, c (Å)	76.57,80.21,85.4	107.04,107.04,360.96	106.98,106.98,363.69	106.84,106.84,364.02
α, β, γ (°)	90,90,90	90,90,90	90,90,90	90,90,90
Resolution (Å)	50.0–2.0	50.0–2.2	50.0–2.4	50.0–2.0
*R*_sym_ (%)^b^	7.6 (25.3)	6.3 (31.5)	6.1 (20.3)	10.2 (20.5)
*I*/σ (*I*)	14.4 (3.1)	19.9 (2.9)	31.1 (3.7)	42.8 (5.16)
Completeness (>1σ,%)	90.0 (70.4)	88.5 (67.1)	87.8 (64.4)	91.3 (77.1)
Redundancy	4.7	3.9	5.8	4.9
Refinement				
Resolution (Å)	50.0–2.0	50.0–2.2	50.0–2.4	50.0–2.0
Number of reflections	32135	95036	73522	124942
*R*_work_ / *R*_free_ (%)	20.8 / 23.8	21.8 / 25.9	20.0 / 24.4	21.9 / 24.9
Number of atoms				
Protein	3929	12087	12196	12198
Water	362	263	312	310
ion	7^c^	17	12	6
R.m.s deviations				
Bond length (Å)	0.006	0.007	0.007	0.006
Bond angles (°)	1.66	1.25	1.26	1.22
Ramachandran plot (%)				
Most favored /Favored	91.4 / 8.1	89.2 / 10.7	88.7 / 11.2	89.8 / 10.1
Generously allowed	0.5	0.1	0.1	0.1
Average B-values (Å^2^)				
Protein	16.3	41.5	43.5	37.4
Water	29.3	37.3	37.1	35.6
ion		38.1	47.3	36.9

^a^Beamline NW12A at Photon Factory; Beamline 5C at Pohang Accelerator Laboratory

^b^The numbers in parentheses are statistics from the highest resolution shell.

^c^Phosphate ions

### Calorimetric analysis of metal ion binding to TON_0340

The affinity of interaction between TON_0340 and metal ions was analyzed by isothermal titration calorimetry (ITC)[13]. All measurements were carried out at 25°C on a MicroCal200 (GE Healthcare). TON_0340 was dialyzed against a solution containing 20 mM Tris-HCl pH 7.5 and 0.1 M NaCl (Buffer B) plus 20 mM EDTA and subsequently against Buffer B at 4°C for 3 h for each dialysis. MnCl_2_, MgCl_2_ or CaCl_2_ was dissolved in Buffer B. The samples were degassed for 10 min and centrifuged to remove any precipitated protein prior to the measurements. The enthalpy changes caused by the injection of each metal ion into buffer were negligible, but these dilution enthalpies were subtracted from the enthalpies of the binding between the protein and the metal ions. The data fitting was performed using the Origin software Version 7.0 (OriginLab Corp.) to deduce the apparent dissociation constant (*K*_D_).

### Phosphatase activity assays

TON_0340 was crystallized in a large scale by mixing 0.1 mL of TON_0340 sample (0.35 mM) and 0.1 mL mother liquor (0.1 M sodium acetate pH 5.5 and 16% MPD) on a nine well glass plate equilibrated with the same mother liquor. Crystals were dissolved and dialyzed against a solution containing 100 mM HEPES pH 7.5 and 100 mM NaCl (Buffer C) plus 2 mM EDTA at 4°C overnight, and subsequently dialyzed against Buffer C. This TON_0340 sample (1 μM) was reacted with each of twenty different phosphate-containing compounds (2 mM) at 37°C in Buffer C and additional 2 mM metal salt (MnCl_2_, MgCl_2_, CaCl_2_ or ZnCl_2_). Various phosphate-containing substrates (2 mM), including adenosine 5’-monophosphate (AMP), were reacted with the apo form of TON_0340 (1 μM) at 37°C for 2 h in a buffer solution containing 100 mM HEPES pH 7.5, 100 mM NaCl and 2 mM MnCl_2_. Released inorganic phosphate (P_i_) was quantified by using either EnzChek phosphate assay kit (Molecular Probes). In this reaction, purine nucleoside phosphorylase phosphorylates 2-amino-6-mercapto-7-methylpurine riboside (MESG) to ribose 1-phosphate and 2-amino-6-mercapto-7-methylpurine whose maximum absorbance is at 360 nm [14]. For reactions involving high Mg^2+^ concentration, SensoLyte MG phosphate assay kit (Anaspec) was used. The blue-green complex formed between malachite green, P_i_ and molybdate was quantified at 630 nm. For determination of the k_cat_/K_M_ value for AMP hydrolysis, reaction velocities were measured with varying concentration of TON_0340. Data were analyzed by fitting them to the pseudo-first order Michaelis-Menten equation

$${\text{A(t) }=\text{ (A}}_{\infty }-{\text{A}}_{0})(1-\text{exp}(-{\text{k}}_{\text{obs}}{\text{ t}))\text{+A}}_{0}$$

where k_obs_ = k_intr_ + (k_cat_/K_M_)[TON_0340]. The k_cat_/K_M_ and intrinsic rate constant (k_intr_) were treated as global parameters.

## Results and Discussions

### Overall structure

**Fig 1:**
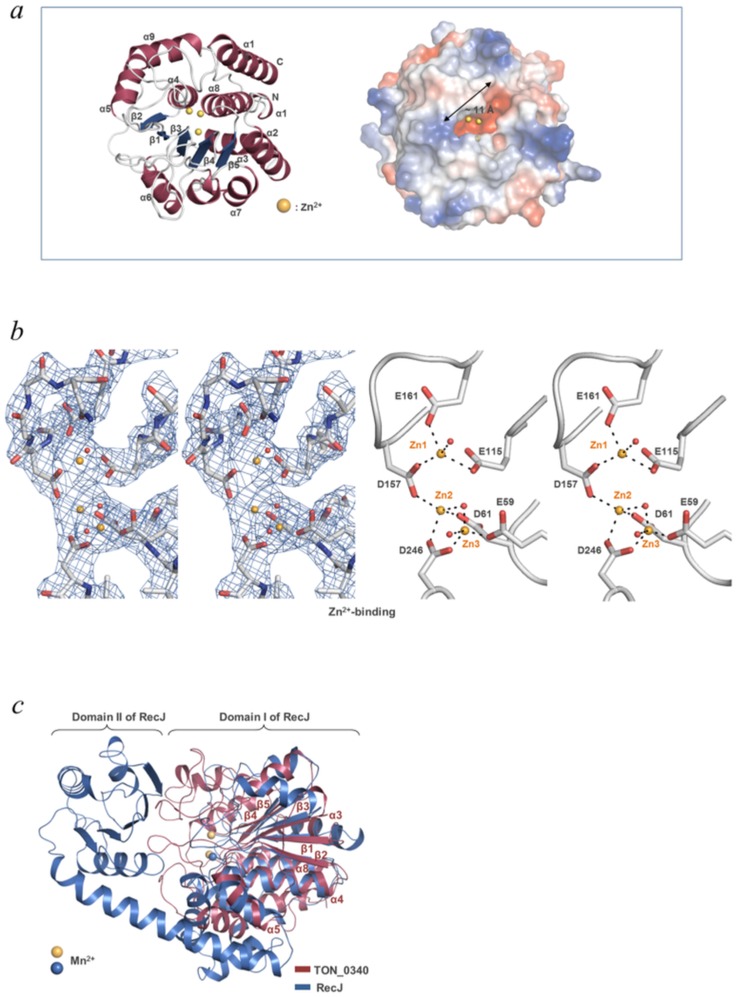
Structural features of TON_0340 monomer. (*a*) Overall structure. On the ribbon drawing (*left*), the secondary structural elements are numbered in the order of the appearance in the primary structure. The surface representation (*right*) highlights the cavity containing the bound Mn^2+^ ions (*spheres*). (*b*) Zn^2+^ ions interacting with a cluster of six acidic residues. The detailed interactions are shown in stereoviews with and without the final 2*F*_o_-*F*_c_ electron density maps (1.0 σ). The bound zinc ions, (Zn1, Zn2, Zn3) are shown as orange spheres, and water molecules as red spheres.(*c*) Structural superposition of TON_0340 and the catalytic core of RecJ (PDB entry: 1IR6). The Cα traces are shown. The superposed α-helices and β-strands in TON_0340 are labeled. The bound Mn^2+^ ions, two in TON_0340 and one in RecJ, are shown in spheres.

Previously, we reported the structure of Zn^2+^-bound TON_0340, but without any description of the structure. We subsequently determined the TON_0340 structures in the apo form and in the Mn^2+^-, Mg^2+^- or Ca^2+^-bound form. The overall structures of TON_0340 in the five different forms are virtually the same. TON_0340 is a globular protein with the overall dimensions of 38 Å x 54 Å x 47 Å. It is composed of ten α-helices and five β-strands that are arranged to form a single-domain structure. The β-strands are all parallel and form a single β-sheet which is surrounded and buried by α-helices and loops (Fig 1a), a folding pattern commonly observed in many different proteins. A surface of the protein has a readily identifiable small cavity. Inside this cavity, closely spaced six acidic residues (Glu59, Asp61, Glu115, Asp157, Glu161, Asp246) are found that interact directly with three Zn^2+^ ions in the Zn^2+^-bound TON_0340 structure (Fig 1b), pointing that the cavity is likely to be an enzyme active site.

Although TON_0340 adopts a common folding pattern, a database search using the program Dali [15] showed that the structure of TON_0340 is not significantly homologous to any known protein structures. The closest match was the structure of the catalytic core domain of the exonuclease RecJ derived from *Thermus thermophilus* (PDB code: 1IR6; Z score = 12.3). The catalytic core (424 amino acids) is composed of two easily discernible domains connected by an α-helix. A structural alignment shows that the central β-sheet and several α-helices of TON_0340 could be grossly superimposed onto those in one of the two domains of RecJ (Fig 1c). Interestingly, the metal-ion binding site of TON_0340 is spatially the same as the catalytic Mn^2+^-binding site in RecJ, whereas TON_0340 has no feature for binding DNA, such as the DNA-binding interdomain cleft present in RecJ [16–18]. The structural comparison did not provide a strong clue about the biochemical function of TON_0340.

### TON_0340 forms a parallel homodimer

**Fig 2:**
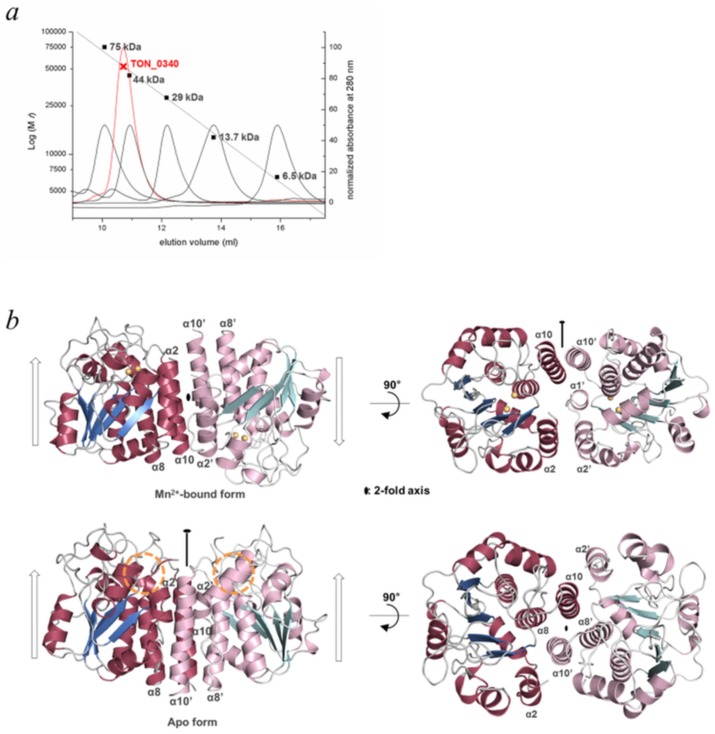
Homodimeric features. (*a*) Estimation of the molecular weight. TON_0340 (100 μM) in Buffer A was loaded on a Superdex 75 10/300 GL analytical column, and eluted at a rate of 0.5 ml/min. The elution profile is shown together with those of the size marker proteins, which were conalbumin (75 kDa), ovalbumin (44 kDa), carbonic anhydrase (29 kDa), ribonuclease A (14 kDa), aprotinin (6.5 kDa).(*b*) Two different homodimeric arrangements in the crystals. Shown are the two TON_0340 molecules forming the crystallographic homodimer in the crystals of Mn^2+^-bound TON_0340 or apo TON_0340. The arrows indicate the relative orientations of the molecules. The circle on the bottom left panel indicates the metal-free clefts.

The molecular weight of TON_0340 deduced from a size-exclusion column chromatography was 52.4 kDa (Fig 2a), which is twice the calculated molecular weight of TON_0340 (29.0 kDa). Consistently, in the asymmetric unit of the Mn^2+^-, Mg^2+^-, Ca^2+^- or Zn^2+^-bound crystals, six molecules of TON_0340 formed three dimeric pairs which are essentially the same with each other, as if this observed homodimer is the biological unit. The two molecules in each pair are in antiparallel orientations (Fig 2b; top panel). The intermolecular interactions, involving α1, α2 and α10, are mostly hydrophobic and quite extensive, burying a surface area of 1,202 Å^2^ in one subunit. Intriguingly, in the crystals of the apo form of TON_0340, two protein molecules in the asymmetric unit also formed a dimer-like pair, but they were in parallel orientations (Fig 2b; bottom panel). The intermolecular interactions are also mostly hydrophobic and bury a surface area of 1,778 Å^2^ in one subunit. The binding interface involves α2, α8 and α10, whereas that in the metal-bound TON_0340 involves α1, α2 and α10 (Fig 2b). The N-terminal segment corresponding to α1 in the metal-bound TON_0340 is disordered in both molecules forming the parallel dimer in the apo form. The helices α8 and α10 of one molecule interact withα8’ and α10’ of the other molecule, as if they form a four-helix bundle (Fig 2b; bottom panel). Probably, the parallel dimer of TON_0340 is the biological unit in solution and the formation of the antiparallel dimer is likely to be caused by the high concentration of 2-methyl-2,4-pentanediol (16%) contained in the crystallization solution that probably disrupted the native hydrophobic interactions at the dimer interface.

### Mn^2+^, Mg^2+^ and Ca^2+^ bind to TON_0340

**Fig 3:**
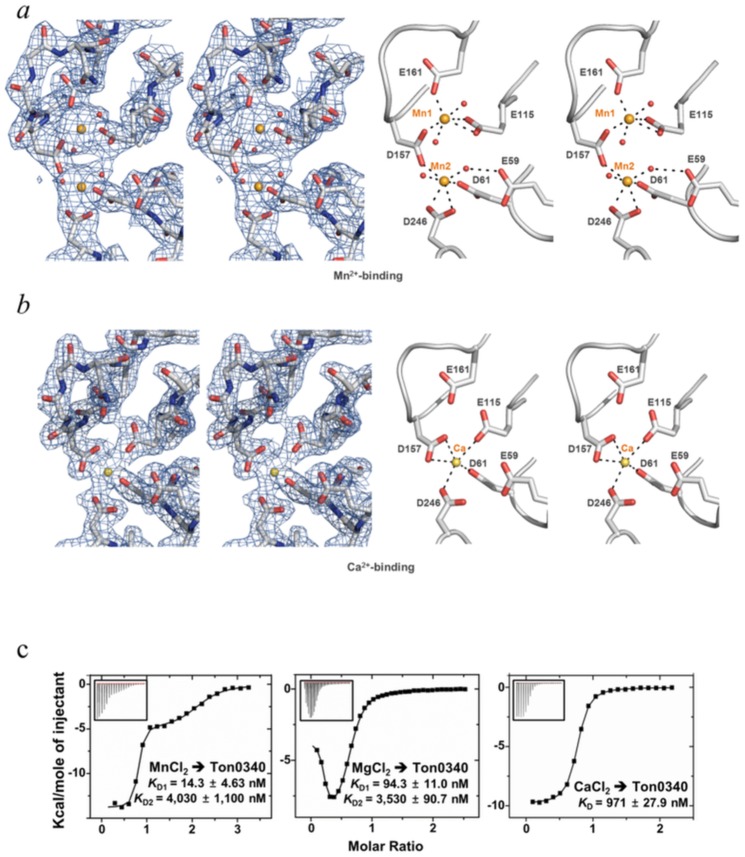
Metal-binding sites of TON_0340. (*a*) Mn^2+^-binding (*b*) Ca^2+^- binding. The detailed interactions between the metal ions and the six closely located acidic residues are shown in the same orientation as in Fig 1b: (*c*) Metal-binding affinity. ITC analysis was carried out by titrating MnCl_2_, MgCl_2_ or CaCl_2_ (1 mM) into TON_0340 (100 μM). The *K*_D_ values were deduced from curve fittings of the integrated heat per mol of the added salt and are shown. *K*_D_(1) and *K*_D_(2) stand for the *K*_D_ for the interaction of the metal ions with the first- and second-binding site of TON_0340, respectively.

We suspected that the binding of Zn^2+^ to the cluster of the six acidic residues might be physiologically irrelevant because Zn^2+^-coordination to a protein usually involves cysteine and/or histidine residue(s), and that Zn^2+^ ions were incorporated nonspecifically due to the high concentration of zinc acetate (350 mM) in the crystallization conditions. In an effort to gain a clue about the physiological metal ligand of TON_0340, we sought to determine the structure of the protein bound to Mn^2+^-, Mg^2+^-, or Ca^2+^, which are usually coordinated by multiple acidic residues in proteins [19, 20]. EDTA-treated TON_0340 was crystallized with the precipitant solution containing MnCl_2_. In this crystal, the cluster of the acidic residues were associated with two prominent but relatively weaker electron densities compared with the Zn^2+^ densities (Fig 3a). These densities were assigned to Mn^2+^ ions. Distinctively from the Zn^2+^-binding mode, two Mn^2+^ ions rather than three Mn^2+^ ions interact directly with five, not six, acidic residues. Glu59, which directly coordinates Zn^2+^, does not participate in the Mn^2+^ coordination. Instead, it is hydrogen-bonded to a water molecule, which axially coordinates Mn^2+^ (Fig 3a). The two Mn^2+^ ions, designated as Mn1 and Mn2, are 4.3 Å apart from each other and bridged by Asp157 that coordinates both of them. Mn1 and Mn2 are both chelated by six coordination arms, two of which are water molecules. We also determined the 2.2 Å resolution structure of Mg^2+^-bound TON_0340, whose crystals grew in the presence of magnesium acetate. The electron densities for two Mg^2+^ ions were visible at the same places that were occupied by Mn^2+^ in the structure of Mn^2+^-bound TON_0340 (not shown), indicating interchangeable binding of Mg^2+^ and Mn^2+^ to the protein. The Ca^2+^-bound TON_0340 structure was determined by growing the crystals in the presence of CaCl_2_. Unexpectedly, only one Ca^2+^ ion bound to the cluster of the acidic residues (Fig 3b). The Ca^2+^ ion was chelated by Glu59, Asp61, Glu115 and Asp157 occupying the Mn2 site. To elaborate these observations further, we quantified the binding of these metal ions to TON_0340 by ITC. Mg^2+^ and Mn^2+^ exhibited two-site interactions with TON_0340, which is consistent with the structural data. The two metal ions interacted with the first site much more tightly than it did with the second site. The deduced apparent dissociation constants (*K*_D_s) for Mn^2+^ were 14 nM and 4.0 μM, whereas those for Mg^2+^ were 94 nM and 3.5 μM (Fig 3c). As anticipated, Ca^2+^ exhibited single-site interaction with TON_0340 with the deduced *K*_D_ of 970 nM. Together, these data suggest that Mn^2+^ is likely to be the physiological metal ion most favored by the clustered acidic residues of TON_0340 in cells.

### High sequence conservation and invariant metal-chelating residues

**Fig 4:**
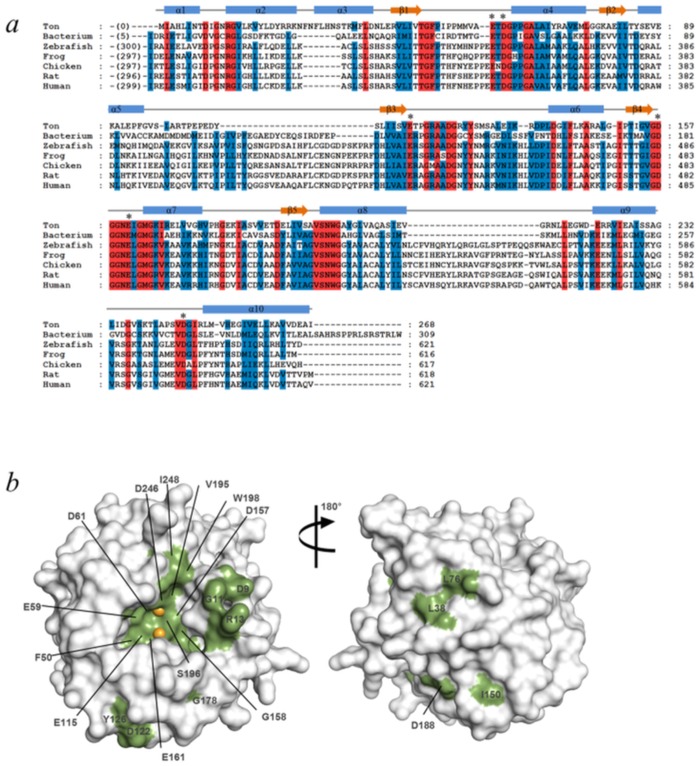
Sequence alignment and conserved residues. (*a*) Multiple sequence alignment. Sequences of TON_0340 and its homologues from seven distant organisms are aligned. The red and blue columns indicate the amino acids that are 100% and greater than 80% conserved, respectively. The metal-binding residues in the TON_0340 structure are indicated by asterisks. The secondary structure assignment is shown at the top of the sequence. The accession numbers in the sequence databases are TON_0340 (GI: 212223486), bacterium (GI: 150392380; Amet_4702 of *Alkaliphilus metalliredigens* QYMF), zebrafish (GI: 115313581), frog (GI: 89272814), chicken (GI: 118092080), rat (GI: 291167745) and human (GI: 31874032; C14orf159). (*b*) Mapping of the invariant residues on the TON_0340 structure. On the surface of the protein, the invariant residues are shown in green and labeled. The bound Mn^2+^ ions are shown in spheres.

A *BLAST* search [21] showed that TON_0340 homologues or homologous domains are present in archaea, bacteria, zebrafish, frog, chicken, platypus, rat and human, but not in fungi, plants and insects. Accordingly, a set of phylogenetically distant TON_0340 homologues were chosen and their sequences were aligned (Fig 4a). The multiple sequence alignment revealed a number of important features. First, TON_0340 is conserved as a domain in a polypeptide comprising two separate domains in higher eukaryotic organisms (from fish). The other domain in these proteins belongs to the DUF1445 superfamily and is homologous to Atu3911 from *Agrobacterium tumefaciens*, a hypothetical protein whose structure is available (PDB ID: 3DB9). Homologues of Atu3911 are found in bacteria, fungi, zebrafish, frog, chicken, rat and human, but not in archaea and plants. Thus, the genes coding for a TON_0340 homologue and an Atu3911 homologue were fused together in higher animals perhaps to perform sequentially linked biochemical functions efficiently. Second, the sequence homology between TON_0340 homologues is notably high (Fig 4a), as exemplified by 36% sequence identity between TON_0340 and the C-terminal domain of human C14orf159, indicating that the biochemical function of TON_0340 might be crucial in many branches of living organisms although it is unnecessary for fungi and plants. Third, the six acidic residues involved in the metal binding are absolutely conserved and most of the surface-exposed invariant residues are concentrated at or near the small cavity (Fig 4), highlighting the importance of the cavity.

### TON_0340 exhibits a phosphatase activity

**Fig 5:**
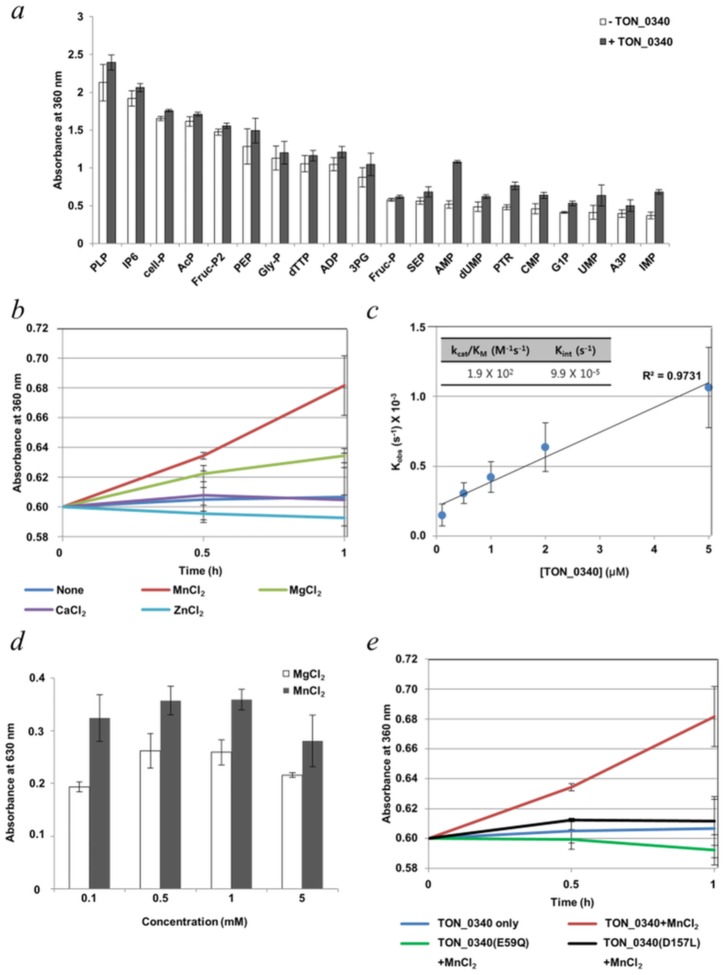
Phosphatase activity of TON_0340. (a) Each of the indicated compounds (2 mM) was incubated with metal-removed TON_0340 (1 μM) at 37°C for 2 h in the presence of 2 mM MnCl_2_. PLP: pyridoxal 5’-phosphate hydrate; IP6: phytic acid sodium salt hydrate; cell-P: cellulose phosphate; AcP: lithium potassium acetyl phosphate; Fruc-P2: D-fructose 1,6-bisphosphate sodium salt; PEP: phospho(enol)pyruvic acid monopotassium salt; Gly-P: sn-glycerol 3-phosphate bis(cyclohexylammonium) salt; dTTP: 2’-deoxythymidine 5’-triphosphate; ADP: adenosine 5’-diphosphate sodium salt; 3PG: D-(-)-3-phosphoglyceric acid disodium salt; Fruc-P: D-fructose 1-phosphate barium salt; SEP: O-phospho-L-serine; AMP: adenosine 5’-monophosphate; dUMP: 2’-deoxyuridine 5’-monophosphate; PTR: O-phospho-L-tyrosine; CMP: cytidine 5’-monophosphate disodium salt; G1P: α-D-glucose 1-phosphate disodium salt; UMP: uridine 5’-monophosphate sodium salt; A3P: adenosine 3’-monophosphate; IMP: inosine 5’-monophosphate disodium salt. (b) Effect of metal ions. Metal-removed TON_0340 (1 μM) was incubated with AMP (2 mM) and the indicated metal ions (2 mM) at 37°C. Inorganic phosphate in the reaction mixture was measured. (c) k_cat_/K_M_ measurement. The reaction mixture containing 2 mM AMP, 2 mM MnCl_2_ and vary concentrations of TON_0340. Production of phosphate was measured in a time-course and used to deduce the k_cat_/K_M_ value. The experiment was performed three times. (d) Mn^2+^ *versus* Mg^2+^. Metal-free TON_0340 (1 μM) was incubated with AMP (2 mM) in the presence of MnCl_2_ or MgCl_2_ (0.1–5 mM) at 37°C for 1 h. Phosphatase activity was measured as in *b* at 0.1–5.0 mM concentration of MnCl_2_ or MgCl_2_ using malachite green. The background absorption of the control was 0.188. (e) Effect of mutations. The E59Q or the D157L mutant of TON_0340 (1 μM) was incubated with AMP (2 mM) in the presence of MnCl_2_ (2 mM) at 37°C for 1 h. Each analysis was performed in triplicates.

The distinct cavity and absolute conservation of the metal-binding residues strongly suggested that TON_0340 is a metal-dependent enzyme. So far, three different oxidoreductases containing a dimanganese center have been identified. One is dimanganese catalase which decomposes H_2_O_2_ [22], another is NrdF, a ribonucleotide reductase [23, 24] and the other is an N-oxygenase AurF which monoxygenates the amino group of *p*-aminobenzoic acid [25, 26]. The dimanganese center in the dimanganese catalase is deeply buried in a narrow channel [22] and that in AurF is encapsulated by α-helices [25, 26], both to create an electron transfer environment. TON_0340 contains a dimanganese center inside a cavity exposed to the bulk solvent (Fig 1a), and thus is unlikely to have either of the two enzyme activities. A dimanganese center also serves as a cofactor in many different phosphatases such as eukaryotic metal-dependent serine/threonine phosphatases [27] and prokaryotic phosphoprotein metallophosphatases [28]. In these enzymes, Mn^2+^ and Mg^2+^ are functionally exchangeable. Since both Mn^2+^ and Mg^2+^ were shown to interact with TON_0340 crystallographically and calorimetrically, we sought to determine whether TON_0340 might have a phosphatase activity. Since the physiological substrate of TON_0340 is unknown, we examined whether the protein might exhibit any phosphatase activity towards phosphate-containing compounds available in the laboratory. A total of 20 different compounds were reacted with TON_0340, and released inorganic phosphate was measured. To rule out contamination of *E*. *coli* phosphatases, the TON_0340 sample used for the activity assay was prepared from dissolved crystals; TON_0340 was crystallized in a large scale and the crystals were dissolved in a buffer solution after extensive wash. Very low but detectable phosphatase activity was observed with a number of compounds in the presence of Mn^2+^ (Fig 5a). The highest activity was observed with AMP, and characterization of the phosphatase activity of TON_0340 was performed with AMP thereafter. The apo-form of TON_0340 exhibited no detectable phosphatase activity. In contrast, TON_0340 exhibited an easily detectable activity in the presence of exogenously added Mn^2+^ (Fig 5b). The catalytic efficiency (k_cat_/K_M_) of TON_0340 for AMP hydrolysis was measured to be 1.9 x 10^2^ M^-1^s^-1^ (Fig 5c).

We found that Mg^2+^ also activates the phosphatase activity, but less efficiently than Mn^2+^ over a wide range of the concentration of the two metal ions. About 2/3 of the enzyme activity in the presence of Mn^2+^ was observed for Mg^2+^ (Fig 5d). Notably, TON_0340 was not activated by the addition of Ca^2+^ or Zn^2+^ (Fig 5b). To test whether the metal-binding site is indeed responsible for the phosphatase activity, we generated a TON_0340 mutant containing a leucine substitution of Asp157 which chelates both Mn1 and Mn2. Thus, this isosteric mutation was a design to disrupt the metal binding. The resulting TON_0340(D157L) mutant lost the phosphatase activity, indicating that the metal-binding site is critical for the catalytic activity (Fig 5e). Like the catalytic metal ions in well-characterized phosphatases, the Mn^2+^ ions bound to TON_0304 are likely to play multiple essential roles: binding the phosphate group of the substrate, stabilizing the transition-state complex and lowering the p*K*a value of bound water molecule. In the Mn^2+^-binding mode, a notable feature is that Glu59 is not involved in the metal-binding, but makes a hydrogen bond to a water molecule that chelates Mn2 (Fig 3a). One possible scenario is that Glu59 functions as a general base that abstracts a proton from the Mn2-chelating water molecule such that the resulting hydroxide ion makes a nucleophilic attack on the phosphate atom of the substrate molecule. To examine whether the carboxylate group of Glu59 is essential for the catalytic activity, we generated a TON_0340 mutant containing a substitution of Glu59 with glutamine. In the presence of Mn^2+^, TON_0340(E59Q) mutant exhibited no detectable phosphatase activity (Fig 5e). Thus, the carboxylate functionality of this invariant residue is critical for the catalytic activity, possibly by playing the role of activating the metal-bound water molecule.

## Conclusions

Our analyses strongly support that TON_0340 is a novel Mn^2+^-dependent phosphatase. The six invariant acidic residues are shown to be involved in binding two metal ions directly or indirectly through a water molecule. Considering the small size of the metal-binding cavity and the conservation of the cavity forming residues, we speculate that the physiological substrate would be a small molecule, and that the TON_0340 homologues are likely to hydrolyze the same substrate molecule. The presented work provides a footstep toward identifying the genuine substrate and also forms an important framework for elucidating the biochemical and biological functions of the TON_0340 homologues found in a variety of living organisms including human.
